# Impact of education on antibiotic literacy and awareness among pharmacy students at a Japanese university: a questionnaire survey

**DOI:** 10.1186/s40780-025-00417-6

**Published:** 2025-02-12

**Authors:** Masayuki Maeda, Kozue Yamaguchi

**Affiliations:** 1 Division of Infection Control Sciences, Department of Clinical Pharmacy, School of Pharmacy, Showa University, 1-5-8 Hatanodai, Shinagawa-ku, Tokyo, 142-8555 Japan; 2 Division of Clinical Infectious Diseases, Department of Infection Control Sciences, Showa University Graduate School of Pharmacy, Tokyo, Japan

**Keywords:** Antibiotic awareness, Antibiotic literacy, Antimicrobial resistance, Antimicrobial stewardship, Pharmacy student

## Abstract

**Background:**

Considering the global threat of antimicrobial resistance (AMR), Japan implemented a national action plan in 2016 that emphasized antibiotic education for healthcare professionals. However, pharmacy education in Japan lacks comprehensive antimicrobial stewardship (AMS) content, leading to insufficient antibiotic awareness and literacy among graduates. This study aimed to assess and improve antibiotic literacy and awareness among undergraduate pharmacy students at Showa University.

**Methods:**

Students who were admitted in 2015 were given a 90-min lecture on AMS, covering acute upper respiratory infections, before their fifth-year clinical training in 2019. The lecture was delivered by a certified pharmacist in infection control. A paper-based, anonymous self-administered questionnaire survey on antibiotic knowledge and literacy was distributed to first- and fifth-year students in 2019 and fifth-year students in 2023. The questions in the survey were based on the Antibiotics Awareness Survey 2018.

**Results:**

The survey results showed an 82–99% response rate among first- and fifth-year pharmacy students in 2019 and 2023. Although two-thirds of first-year pharmacy students lacked antibiotic knowledge, most fifth-year students had appropriate knowledge. Moreover, fifth-year students had a proper understanding of bacterial infections and antibiotic identification, which improved after clinical training and additional lectures. The percentage of students who would stop using antibiotics when they felt better dropped from 39% among first-year students to 21% among fifth-year students. Fifth-year students were more likely to dispose of leftover antibiotics and less likely to keep them than first-year students. Over 80% of students provided antibiotic counseling primarily for common cold treatments during clinical training.

**Conclusions:**

The survey results highlighted gaps in antibiotic awareness among Japanese pharmacy students and demonstrated the positive impact of education. This study emphasizes the need for an antibiotic literacy curriculum, especially for viral respiratory infections, to support efforts in curbing AMR. Moreover, policymakers should develop policies for developing and securing faculty that are knowledgeable in clinical infectious diseases across Japanese pharmacy schools.

## Background

Antimicrobial resistance (AMR) has been a major public health threat worldwide. Based on the Global Action Plan on Antimicrobial Resistance, Japan implemented a national action plan (NAP) on AMR in 2016 [1]. One aim of this NAP is to promote smart antibiotic choices by educating healthcare professionals. As a specific policy of the NAP, the Ministry of Health, Labour and Welfare issued guidance on the appropriate use of antibiotics for acute upper respiratory infections in 2017, including the common cold [2]. This guidance primarily targets physicians who prescribe antibiotics. Since then, Japan’s overall antibiotic usage has been decreasing annually [3–5]. However, although the Japanese government has implemented policies aimed at increasing antibiotic literacy among the general public, the effectiveness of these initiatives remains limited [6].

In Japan, 6 years of undergraduate education is required to become eligible for the National Examination for Pharmacists. In the Model Core Curriculum for Pharmacy Education established by the Ministry of Education, Culture, Sports, Science and Technology, first- to fourth-year pharmacy students acquire knowledge of basic pharmaceutical sciences, including microbiology, pharmacology, and pathology by organs [7, 8]. Afterward, fifth-year pharmacy students undergo a 22-week clinical practice at a hospital and community pharmacy. However, the contents of antimicrobial stewardship (AMS) have not been well incorporated into the curricula of pharmaceutical schools [8]. Pharmacists play an important role in promoting the appropriate use of antibiotics in hospitals and community pharmacies by educating doctors and patients [9–12]. Nevertheless, there is a serious concern regarding a shortage of faculty members who have expertise in clinical infectious diseases at Japanese pharmaceutical schools [13]. Therefore, most pharmacy students might graduate without sufficient antibiotic literacy. Previous surveys that focused on Japanese medical and pharmacy students revealed that undergraduates had inaccurate knowledge and inadequate literacy related to the use of antibiotics [14, 15]. Therefore, in the present study, we provided education on antibiotic knowledge and literacy for undergraduate pharmacy students and aimed to evaluate the current status of antibiotic literacy and education outcomes among Japanese pharmacy students.

## Methods

### Study setting and design

This study was conducted on undergraduate students of Showa University School of Pharmacy. In our university, pharmacy students take lectures on microbiology and infectious diseases during the second to third year according to the Model Core Curriculum for Pharmacy Education in Japan [8]. During this period, the education focuses on basic knowledge of infectious diseases and antibiotics and does not include content on antibiotic literacy and awareness. Students who were admitted in 2015 received education on antimicrobial literacy before their fifth-year clinical training in 2019. Based on the Manual of Antimicrobial Stewardship published by the Ministry of Health, Labour and Welfare [2], a 90-min lecture on acute upper respiratory tract infections (e.g., common cold, pharyngitis, sinusitis) was conducted. The lecturer (M.M.) is a Board Certified Infection Control Pharmacy Specialist and Infectious Diseases Chemotherapy Pharmacist.

### Questionnaire

A paper-based, anonymous self-administered questionnaire survey comprising questions related to antibiotic knowledge and literacy was administered to first- and fifth-year students in 2019 and fifth-year students in 2023. The survey items and questions (Q1 to Q7) were set based on the Antibiotics Awareness Survey 2018, which was conducted on Japanese citizens [16]. The questions and choices in this questionnaire are shown in Tables 1, 2 and 3. As a primary outcome of the additional lecture on acute upper respiratory infections, improvement of antibiotic literacy in recognizing that “antibiotics are effective against the common cold” and “antibiotics are necessary for the common cold” was evaluated using Q3 and Q5.

**Table 1:** Percentage of response choices regarding antibiotic knowledge and awareness among first- and fifth-year pharmacy students

Question and choices	First-year in 2019	*Lecture cohort*		*Non-lecture cohort*	
		Fifth-year in 2019 (preclinical training and lecture)	Fifth-year in 2019 (postclinical training and lecture)	Fifth-year in 2023 (preclinical training)	Fifth-year in 2023 (postclinical training)
Q1: Do you know the term “antibiotics”?					
Can be explained to others	2 (1.0)	29 (19.9)	63 (36.8)	47 (27.6)	61 (38.1)
Yes	64 (31.8)	117 (80.1)	106 (62.0)	120 (70.6)	99 (61.9)
No	135 (67.2)	0	2 (1.2)	3 (1.8)	0
Q2: What are the effect of antibiotics?					
Break a fever	37 (18.5)	20 (13.7)	26 (15.2)	24 (14.1)	22 (13.8)
Relieve pain	27 (13.5)	7 (4.8)	9 (5.3)	16 (9.4)	10 (6.3)
Inhibit bacterial growth	182 (91.0)	141 (96.6)	169 (98.8)	161 (94.7)	156 (97.5)
Stop coughing	29 (14.5)	8 (5.5)	10 (5.8)	10 (5.9)	9 (5.6)
Control runny nose	27 (13.5)	11 (7.5)	11 (6.4)	11 (6.5)	9 (5.6)
Suppress inflammation	80 (40.0)	42 (28.8)	50 (29.2)	53 (31.2)	56 (35.0)
Q3: Do you know what kind of diseases antibiotics are effective?					
Common cold	91 (45.0)	61 (41.8)	34 (19.9)	74 (43.5)	70 (43.8)
Influenza	93 (46.6)	20 (13.7)	18 (10.5)	25 (14.7)	9 (5.6)
Cystitis	32 (15.8)	103 (70.5)	149 (87.1)	91 (53.5)	128 (80.0)
Pneumonia	52 (25.7)	113 (77.4)	154 (90.1)	115 (67.6)	120 (75.0)
Otitis media	23 (11.4)	103 (70.5)	156 (91.2)	108 (63.5)	113 (70.6)
Norovirus	94 (46.5)	9 (6.2)	9 (5.3)	19 (11.2)	7 (4.4)
Diarrhea	24 (11.9)	28 (19.2)	39 (22.8)	28 (16.5)	29 (18.1)
Others and no answer	8 (4.0)	6 (4.1)	5 (2.9)	1 (0.6)	1 (0.6)
Q4: Which medication is an antibiotic?					
Oseltamivir	52 (25.7)	26 (17.8)	12 (7.0)	33 (19.4)	13 (8.1)
Amoxicillin	33 (16.3)	121 (82.9)	163 (95.3)	138 (81.2)	152 (95.0)
Pravastatin	36 (17.8)	3 (2.1)	0	2 (1.2)	3 (1.9)
Cisplatin	23 (11.4)	3 (2.1)	0	5 (2.9)	1 (0.6)
Levofloxacin	39 (19.3)	121 (82.9)	161 (94.2)	128 (75.3)	147 (91.9)
Indomethacin	43 (21.3)	5 (3.4)	2 (1.2)	9 (5.3)	9 (5.6)
Chlorpheniramine	41 (20.3)	2 (1.4)	9 (5.3)	16 (9.4)	8 (5.0)
Paroxetine	14 (6.9)	3 (2.1)	0	9 (5.3)	1 (0.6)
No answer	33 (16.3)	3 (2.1)	1 (0.6)	0	0
Q5: What kind of medication do you think is necessary for a patient with a common cold?					
Antitussive	186 (92.1)	130 (91.5)	153 (90.0)	145 (85.3)	146 (91.3)
Antipyretic	138 (68.3)	119 (83.8)	144 (84.7)	142 (83.5)	147 (86.5)
Antibiotics	69 (34.2)	36 (25.4)	24 (14.1)	44 (25.9)	45 (26.5)
Lozenge/troches	83 (41.1)	53 (37.3)	101 (59.4)	70 (41.2)	97 (57.1)
Expectorant	108 (53.5)	110 (77.5)	158 (92.9)	122 (71.8)	134 (78.8)
Analgesic	13 (6.4)	28 (19.7)	60 (35.3)	26 (15.3)	45 (26.5)
Gargle	57 (28.2)	53 (37.3)	92 (54.1)	51 (30.0)	75 (44.1)
Stomach medicine	34 (16.8)	17 (12.0)	47 (27.6)	15 (8.8)	44 (25.9)
Others	1 (0.5)	2 (1.4)	4 (2.4)	0	1 (0.6)

Data are presented as the number of valid responses (%)

Multiple answers were allowed for Q2–Q5

**Table 2:** Percentage of responses to questions regarding antibiotic literacy among first- and fifth-year pharmacy students

Question and choices	First-year in 2019	Fifth-year in 2019 (preclinical training)	Fifth-year in 2023 (preclinical training)
Q6: Do you finish taking a full course of antibiotics when they are prescribed to you?			
I complete the full course of antibiotics	74 (37.4)	84 (58.3)	98 (57.6)
I discontinue antibiotics once I feel recovered	77 (38.9)	30 (20.8)	35 (20.6)
I forget to take them and have not used them all	12 (6.1)	22 (15.3)	19 (11.2)
I minimize my use of antibiotics	4 (2.0)	4 (2.8)	4 (2.4)
I have never taken antibiotics	31 (15.7)	4 (2.8)	14 (8.2)
Q7: What do you do with leftover antibiotics?			
I save them for later use	74 (37.0)	60 (42.0)	51 (30.0)
I take them when I feel sick	31 (15.5)	10 (7.0)	14 (8.2)
I give them to my family or friends	1 (0.5)	0	1 (0.6)
I dispose them all	43 (21.5)	46 (32.2)	61 (35.9)
I never have any leftovers/I have never taken antibiotics	51 (25.5)	27 (18.9)	41 (24.1)

Data are presented as the number of valid responses (%)

Multiple answers were allowed for Q6–Q7

**Table 3:** Survey results on antibiotic medication counseling during clinical training at the community pharmacy

Question and choices	Fifth-year in 2019 (postclinical training and lecture)	Fifth-year in 2023 (postclinical training)
Q8-1: Did you provide medication counseling for antibiotics during clinical training at the community pharmacy?		
Yes	149 (87.1)	133 (83.1)
Q8-2: If so, for which conditions did you provide this counseling?		
Common cold	74 (49.7)	71 (53.4)
Tooth extraction	58 (38.9)	35 (26.3)
Sinusitis	51 (34.2)	38 (28.6)
Pharyngitis	45 (30.2)	38 (28.6)
Otitis media	44 (29.5)	26 (19.5)
Cystitis	40 (26.8)	30 (22.6)
Prophylaxis for infections	29 (19.5)	26 (19.5)
Pneumonia	17 (11.4)	9 (6.8)
Diarrhea	7 (4.7)	9 (6.8)
Influenza	3 (2.0)	10 (7.5)
No recollection	3 (2.0)	9 (6.8)
Unknown	1 (0.7)	0
Norovirus	0	1 (0.8)
Others	20 (13.4)	10 (7.5)

Data are presented as the number of valid responses (%)

Multiple answers were allowed for Q8-2

### Statistics

The survey results were entered into Microsoft Excel, and data summarization was performed using SPSS Statistics version 26.0 (IBM Japan, Tokyo, Japan). Statistical analyses were not conducted in this study because of the large number of survey items and the inclusion of multiple comparison groups. There was a concern regarding an increase in type I errors from performing repeated statistical tests.

### Ethics

The study was approved by the research ethics committee of Showa University (Approval no. 349 and 22-192-B). An overview of the survey was clearly explained at the beginning of the questionnaire, and a consent checkbox for obtaining consent to use the data in the study was provided.

## Results

### Survey results and fundamental knowledge of antibiotics among first- and fifth-year pharmacy students

For the survey conducted in 2019, the response rates were 99.0% (202/204) in May among first-year students, 82.0% (146/178) in April among fifth-year students before clinical training, and 96.6% (171/177) in November among fifth-year students after clinical training. Meanwhile, for the survey conducted in 2023, the response rates were 87.6% (170/194) in January among fifth-year students before clinical training and 86.5% (160/185) in November among fifth-year students after clinical training.

The survey results regarding pharmacy students’ knowledge and awareness of antibiotics are shown in Table 1. Although two-thirds of first-year students responded that they did not know about antibiotics, almost all fifth-year students responded that they were familiar with them, with over one-third stating that they had sufficient knowledge to explain antibiotics to others. Although 91% of first-year students correctly answered that antibiotics inhibit bacterial growth, nearly half of them incorrectly believed that antibiotics are effective against viral infections such as the common cold, influenza, and norovirus (Table 1, Q2 and Q3). Moreover, they had difficulty in identifying antibiotics based on drug names (Table 1, Q4). However, most fifth-year students answered that antibiotics suppress bacterial growth, and a greater number of fifth-year students responded that antibiotics are effective for bacterial infections such as cystitis, pneumonia, and otitis media compared with first-year students (Table 1, Q2 and Q3). Furthermore, the number of fifth-year students who could correctly identify antibiotics such as amoxicillin and levofloxacin by their names increased (Table 1, Q4). This trend became even more pronounced after clinical training. One-third of first-year students answered that antibiotics are necessary for patients with the common cold, but this decreased to one-quarter among fifth-year students (Table 1, Q5). In addition, the percentage of students who believed that antibiotics were necessary for the common cold was lower in the group that had attended a lecture on the appropriate use of antibiotics for acute upper respiratory infections than in the group that did not (14.1% vs. 26.5%; Table 1, Q5).

### Antibiotic literacy, awareness, and attitude among first- and fifth-year pharmacy students

The survey results on antibiotic literacy, awareness, and attitude among pharmacy students are shown in Table 2. The percentage of students who answered that they would complete the full course of antibiotics was 37% among first-year students and 58% among fifth-year students (Table 2, Q6). Furthermore, the percentage of students who responded that they would stop taking antibiotics once they felt better decreased from 39% among first-year students to 21% among fifth-year students. Although more fifth-year students reported forgetting to take their antibiotics than first-year students, 15.7% of first-year students stated that they had never taken antibiotics. The percentage of students who answered that they would keep leftover antibiotics was 37% among first-year students and 30–42% among fifth-year students (Table 2, Q7). However, the percentage of students who answered that they would take antibiotics when they felt sick was lower among fifth-year students than among first-year students. Finally, the percentage of students who responded that they would dispose of all leftover antibiotics was higher among fifth-year students than among first-year students.

### Survey on the provision of antibiotic medication counseling during clinical training at the community pharmacy

The survey results on antibiotic medication counseling during clinical training at the community pharmacy are shown in Table 3. Over 80% of students answered that they provided counseling on antibiotic use (Table 3, Q8-1). The most common situation was counseling for antibiotics prescribed for the common cold, with about half of students answering (Table 3, Q8-2). This was followed by counseling for tooth extraction, sinusitis, pharyngitis, otitis media, and cystitis. The trend was similar in 2019 and 2023.

## Discussion

The present study revealed the impact of education on antibiotic literacy and awareness among pharmacy students at a Japanese University. The basic education curriculum on microbiology and the pathology and pharmacotherapy of infectious diseases in pharmacy school improved the retention of knowledge regarding antibiotics. Furthermore, students’ understanding of bacterial and viral infections and their awareness of antibiotic use for these diseases were improved by adding lectures on the appropriate use of antibiotics and acute upper respiratory infections. Previous studies found that medical and pharmacy students in Japan had a low antibiotic literacy [14, 15]. To the best of our knowledge, this study is the first to clarify the effectiveness of an antibiotic literacy education curriculum in a Japanese pharmacy school.

In a previous study focusing on medical students, over 90% of students from first to sixth year recognized that antibiotics inhibit bacterial growth, which is consistent with the results of this study [14]. Although not addressed in the previous study, the proportion of fifth-year students that selected other indirect effects was decreased compared with that of first-year students in the present study, suggesting that correct knowledge had been improved. Moreover, more than 80% of fifth-year students could correctly identify antibiotics based on the drug names. In the present study, 45% of first-year students recognized that antibiotics are effective for the common cold, and this percentage hardly decreased among fifth-year students who did not receive additional lectures. In previous studies, approximately 39.4% of fourth-year pharmacy students [15] and 23.8% of fifth-year medical students recognized that antibiotics are effective for the common cold [14]. However, in the present study, the percentage decreased to 19.9% among students who received additional lectures. Moreover, the proportion of students who answered that antibiotics are necessary for patients with the common cold was 34.2% among first-year students, 25.4–26.5% among fifth-year students, and 14.1% among students who had attended additional lectures. These results emphasize the importance of conducting literacy education on the unnecessary use of antibiotics for the common cold separately from microbiology lectures.

In this study, 46% of first-year students recognized that antibiotics were effective against viral diseases such as influenza and norovirus. However, this figure decreased to 10% among fifth-year students. Moreover, the percentage of students who correctly identified that antibiotics are effective for bacterial infections such as cystitis, pneumonia, and otitis media was 20% among first-year students and 70% among fifth-year students, which increased to 80–90% after clinical training. In a previous study on fourth-year pharmacy students, approximately 30% of students believed that antibiotics were effective for influenza and norovirus, whereas 40–60% of students recognized their effectiveness for cystitis, pneumonia, and otitis media [15]. This difference may be because of variations in the education curriculum on pathology and pharmacotherapy between pharmacy schools. A study conducted in 12 pharmacy schools in the USA also reported variations in students’ knowledge and understanding of antibiotics across different schools [17]. Furthermore, most pharmacy students in the study recognized that having a strong knowledge of antibiotics is important for their careers as pharmacists and expressed a desire for more education on the appropriate use of antibiotics. Efforts to share best practices in infectious diseases education and minimize variations between educational institutions would be necessary [18]. Therefore, we suggest the integration of the content of the Guidelines for the Appropriate Use of Antimicrobials developed by the Ministry of Health, Labour and Welfare into the pharmacy educational curriculum. Moreover, this study indicated that even lecture-based passive education by instructors specializing in infectious diseases can be effective for acquiring knowledge of AMS. The use of active learning approach–based educational programs to teach AMS topics was reported to be effective [19]. Considering that antibiotic literacy and AMS do not necessarily require advanced specialized knowledge, implementing an active learning program that is relevant to antibiotic literacy and AMS during the early years of pharmacy education may be effective.

In previous studies investigating the literacy regarding prescribed antibiotics [15, 16], approximately 30% of the general public and 20% of fourth-year pharmacy students reported that they would stop taking antibiotics once their symptoms improved, which is consistent with the results in the present study. A study conducted on medical students reported that approximately 30% of students from first to sixth year responded that they would keep leftover antibiotics, and approximately 20% responded that they would take antibiotics when they felt sick [14]. Interestingly, these inappropriate attitudes in medical students increased with the school year. In the present study, the percentage of students who would keep leftover antibiotics did not change, whereas the proportion of students who would take antibiotics when they felt sick decreased among fifth-year students. Furthermore, the percentage of students who responded that they would dispose of all leftover antibiotics increased among fifth-year students than among first-year students. Previous surveys from different geographical areas have also indicated that education on AMR, antibiotic literacy, and AMS among pharmacy students was insufficient [20, 21]. These results highlight the need for literacy education on the appropriate use of not only antibiotics but also various medications for students in the healthcare field.

In Japan, pharmacy students need to complete clinical training at community pharmacies [7]. These pharmacies are private institutions, not public, and the characteristics of attending patients that students work with vary depending on the training pharmacy. The present study revealed the types of diseases for which pharmacy students provide medication counseling on antibiotics during their clinical training. The training was mainly conducted from May to October, and more than 80% of students responded that they had experience providing medication counseling on antibiotics. Notably, approximately 50% of students responded that they provided counseling on antibiotics to patients with the common cold. A survey targeting Japanese clinic physicians reported that approximately 60% of doctors rarely prescribe antibiotics for the common cold, and this percentage has been increasing over the years [13, 22, 23]. Furthermore, antibiotic prescriptions for upper respiratory infections have reportedly been decreasing over the years [4, 24]. Since antibiotics can only be prescribed by doctors in Japan, it would be challenging for pharmacy students to intervene in common-cold-related antibiotic prescriptions. It is not an ideal situation that half of the students’ experience providing antibiotic counseling for the common cold. In Japan, policies aimed at promoting the appropriate use of antibiotics for acute respiratory tract infections, which primarily target physicians in clinics, have been implemented [2, 3, 23–25]. However, it would take time to confirm the effects of these policies. However, approximately 30% of students gained experience in providing medication counseling for common bacterial diseases, including sinusitis, pharyngitis, otitis media, and cystitis. Although these minor illnesses may not require an extensive educational curriculum, education on these infections would be crucial for training pharmacy students.

The present study has several limitations. First, this survey was conducted at a single institution. Additionally, it should be noted that the educational outcomes observed in this study were based on data from fifth-year students. Therefore, the results cannot be generalized. Japanese pharmacy schools are reportedly facing a shortage of faculty specializing in clinical infectious diseases [13], and there are significant differences in the implemented curricula. The results of this study highlight the need for a standardized infectious disease education system in pharmacy schools. Second, this survey was a self-reliance questionnaire survey, which may include bias. Third, to estimate the true educational effect, direct comparison of groups within the same cohort between those who received and those who did not receive educational intervention would have been ideal. However, because of the educational curriculum and policies of the university that require provision of the same content to all students in the same academic year, such a study design was challenging to implement. In the future, adopting interventional study designs to evaluate the educational method would be necessary to consider. Finally, the response rate for the survey conducted on fifth-year students in 2019 before the lectures was lower compared with that of others. Because this survey was conducted before the lectures, students who were late or absent from the lectures were excluded. As a result, the effect of additional lectures might have been underestimated because of the absence of these students.

## Conclusions

This study revealed the status of antibiotic awareness and literacy among pharmacy students in Japan and demonstrated that these aspects could be improved by providing education. This study reinforces the importance of providing education on antibiotic stewardship, especially for acute upper respiratory infections such as the common cold. The implementation of an antibiotic literacy curriculum across all pharmacy schools in Japan would be important for promoting AMR countermeasures and the appropriate use of antibiotics. Moreover, policymakers and stakeholders should consider creating policies for developing and securing faculty that are knowledgeable in clinical infectious diseases in Japanese pharmacy schools.

## Data Availability

Data available on request due to restrictions (e.g., privacy or ethical).

## Abbreviations

AMS: Antimicrobial stewardship
AMR: Antimicrobial resistance
NAP: National action plan
